# Skyrmion Creation and Manipulation by Nano-Second Current Pulses

**DOI:** 10.1038/srep22638

**Published:** 2016-03-03

**Authors:** H. Y. Yuan, X. R. Wang

**Affiliations:** 1 School of Microelectrics and Solid-State Electronics, University of Electronic Science and Technology of China, Chengdu, Sichuan 610054, China; 2 Physics Department, The Hong Kong University of Science and Technology, Clear Water Bay, Kowloon, Hong Kong; 3 HKUST Shenzhen Research Institute, Shenzhen 518057, China

## Abstract

Easy creation and manipulation of skyrmions is important in skyrmion based devices for data storage and information processing. We show that a nano-second current pulse alone is capable of creating/deleting and manipulating skyrmions in a spin valve with a perpendicularly magnetized free layer and broken chiral symmetry. Interestingly, for an in-plane magnetized fixed layer, the free layer changes from a single domain at zero current to a Neel wall at an intermediate current density. Reverse the current polarity, the Neel wall changes to its image inversion. A properly designed nano-second current pulse, that tends to convert one type of Neel walls to its image inversion, ends up to create a stable skyrmion without assistance of external fields. For a perpendicularly magnetized fixed layer, the skyrmion size can be effectively tuned by a current density.

Skyrmions, originally proposed in nuclear physics to describe resonance states of baryons^1^, were unambiguously observed in many magnetic systems which break the inversion symmetry^2^,^3^,^4^,^5^,^6^,^7^,^8^,^9^,^10^,^11^. One type of such systems such as MnSi, Fe_1−*x*_Co_*x*_Si, Cu_2_OSeO_3_^3^,^4^,^5^,^6^ with the bulk Dzyaloshinskii-Moriya interaction (DMI), has chiral crystal structure that supports vortex-like skyrmions. Another type of the systems are heavy metal/ultrathin ferromagnetic layers such as Fe/Ir, Ni/Co and Ta/CoFeB/TaO_*x*_^10^,^11^,^28^,^29^, that results in an interfacial DMI. Different from the first type of systems, the second type of systems favors hedgehog-like skyrmions. Due to its small size (order of 1 nm–100 nm) and low driven current density^12^ (as low as 10^6^ A/m^2^) in comparison with order of 10^12^ A/m^2^ for a magnetic domain wall, magnetic skyrmions are believed to be potential information carriers in future high density data storage devices and information processing devices^2^,^3^,^4^,^5^,^6^,^7^,^8^,^9^,^10^,^11^,^12^,^13^,^14^.

Easy creation and manipulation of skyrmions is apparently important in these applications. Though isolated skyrmions and skyrmion lattices have been observed in experiments, controlled and effective creation and/or manipulation of skyrmions is still a challenge. The stray field of a hard-magnetized tip was used to create skyrmions in perpendicularly magnetized disks^7^,^15^ by reversing magnetization at the disk center. Scanning tunneling microscope tips were also used to write and delete individual skyrmions in ultrathin magnetic films^8^. Recently, an artificial skyrmion was created by embedding a magnetic vortex into an out-of-plane aligned spin environment^9^. Skyrmions can also be generated by pushing either transverse domain walls^16^ or domains^11^ through geometrical constrictions. From the application viewpoint, the requirement for skyrmion creation should not be too demanding. An easy and efficient method for creating and manipulating skyrmions is essential in spintronic applications, and this is the focus of current work.

In this paper, we show that a current can affect the states of a perpendicularly magnetized circular thin disk via spin-transfer torque (STT). In the context of the thin free layer in a spin valve, the STT induced by a tunneling current is controlled by both the magnetization of the fixed layer and current polarity. For an in-plane magnetized fixed layer, the STT tends to pull spins in the original perpendicularly magnetized free layer into the plane. The ground state of the disk is a single domain and hedgehog skyrmion is a metastable state in the absence of the STT. The single domain transforms into a Neel wall at an intermediate current density. Reverse the current polarity, the Neel wall changes to its image inversion. The magnetization of the disk prefers single domain state with all spins lying in the plane when the current density is larger than a critical value. Interestingly, under a nano-second current pulse whose STT tends to switch a Neel wall to its image inversion, a skyrmion can be created from and deleted to a single domain if the pulse heights and duration are properly controlled. For perpendicularly magnetized fixed layer from which current-induced STT prefers the spins perpendicular to the disk, the size of a metastable skyrmion can be controlled by a current.

Generally speaking, transformation between two spin textures^17^,^18^ in a system can only occur when both textures are stable or metastable. This is also the case for skyrmion creation in spin valves. One interesting issue is what kind of spin textures can normally coexist with skyrmions, and what kind of external forces is desirable and efficient in triggering the creation of a skyrmion from a non-skyrmion state such as a single domain or Neel wall shown below. Previous studies^2^,^19^ showed that perpendicularly magnetized magnetic films with DMI support skyrmion in the absence of an external field although the ground states are single domains. It is noticed that the spin structure along the radial direction of a hedgehog skyrmion is very similar to that of a Neel wall^11^. Thus, Neel wall and skyrmion may have certain connections with each other although they have different skyrmion numbers^12^,^20^:


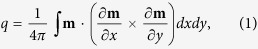


where **m** is the unit vector of local magnetization and *x*, *y* lie on the magnetic film plane; *q* = 0 for a Neel wall and a single domain while *q* = ±1 for a hedgehog-like skyrmion. Different topological numbers of a skyrmion and a Neel wall or a domain mean that they are topologically inequivalent, and they cannot continuously deform from one into the other without involving sample edges. Thus, the skyrmion could only be created from the system edge if one wants to use a current pulse to create it out of a Neel wall or a single domain involving topological change. Naturally, one would like to consider spin valves because they are the basic architectures of many spintronic devices as well as their small size with well defined boundary. Also, the spin valves naturally have the interfacial DMI in its free layer where one wants to create a skyrmion on-demand when the spacer layer is made of certain heavy metals that breaks inversion symmetry.

The spin valves, which we considered here, are nanopillars as shown in Fig. 1. The free layer is an ultrathin circular Cobalt layer of 0.4 nm thick and 80 nm in diameter. A non-magnetic spacer layer separates the free layer from a ferromagnetic fixed layer. The free layer is perpendicularly magnetized when the spacer is heavy metal such as Pt^21^. There exists an interfacial DMI of the form 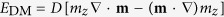, where *D* and **m** are the DMI strength and the unit direction of the magnetization of the free layer, respectively. The free layer lies in *x* − *y* plane and layer normal is along *z*-axis. The magnetization **m**_*p*_ of the fixed layer is along either +*x*-direction (Fig. 1(a)) or +*z*-direction (Fig. 1(b)). The dynamics of the magnetization **m** of the free layer is governed by the Landau-Lifshitz-Gilbert (LLG) equation,


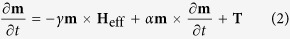


where *γ* and *α* are respectively the gyromagnetic ratio and the Gilbert damping constant. The effective field 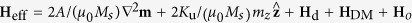 includes exchange field, crystalline anisotropy field, dipolar field, DMI field^21^ and the Oersted field generated by the electric current. The material parameters of the ultrathin Cobalt^22^ are *A* = 1.5 × 10^−11^ J/m, *M*_*s*_ = 5.8 × 10^5^ A/m, anisotropy coefficient *K*_u_ = 8.0 × 10^5^ J/m^3^ and *α* = 0.3. The interfacial DMI is assumed to be uniform across the film normal direction^21^ and the DMI strength *D* is taken to be 3 mJ/m^2^. The third term **T** is STT due to electric current that includes a Slonczewski torque^23^ and a field-like torque,


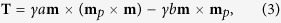


in which


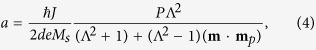



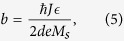


where *ħ*, *J*, *d*, *e*, *M*_*s*_, *P*, є are respectively the Plank constant, current density, the free layer thickness, the electron charge, the saturation magnetization, polarization of conduction electrons, and the coefficient of field-like torque. For Cobalt, Λ = 2.0, *P* = 0.2 and є= 0.2^22^. Electrons flow along +*z* (−*z*) directions for *J* < 0 (*J* > 0). The LLG equation is numerically solved by MUMAX^3^ package^24^. The mesh size is 1 × 1 × 0.4 nm^3^. We have carried out simulations with and without the Oersted field and found that the Oersted field makes neglectable contribution. Therefore, the Oersted field will not be included in the following discussions.

## Results and Discussion

**Figure 2:**
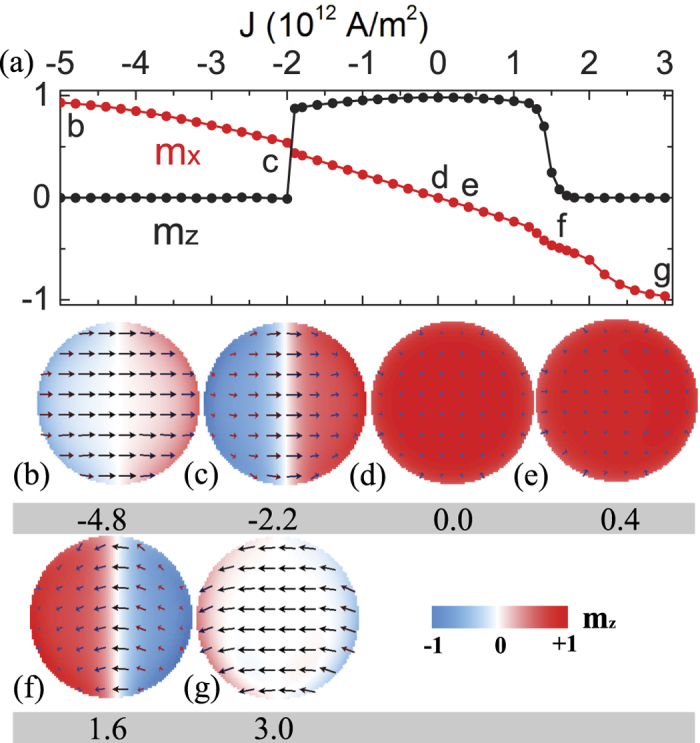
Spin configurations at various current densities. (**a**) Average *m*_*x*_ and *m*_*z*_ as a function of current density. (**b**–**g**) Spin configurations at *J* = −4.8 × 10^12^ A/m^2^, −2.2 × 10^12^ A/m^2^, 0.0, 0.4 × 10^12^ A/m^2^, 1.6 × 10^12^ A/m^2^ and 3.0 × 10^12^ A/m^2^. The current density in units of 10^12^ A/m^2^ is indicated at the bottom of corresponding configuration. The current is switched on throughout the simulations. The arrows are the projection of **m** into the xy-plane. The color bar coding of **m**_*z*_ is shown at bottom-right.

Case of **m**_*p*_ = (1, 0, 0). In the absence of STT, the ground state of the free layer is a perpendicularly magnetized single domain in which all spins are approximately along either +*z*- or −*z*-direction, as shown in Fig. 2(d), where the arrows are the projection of unit vectors of magnetization in the *xy*-plane. One can see that spins at the circular boundary tilt slightly inward due to the DMI^22^. A hedgehog skyrmion is a metastable state that is topologically protected because the skyrmion number cannot be destroyed in a continuous transformation unless the skyrmion annihilates into the sample boundary.

In the case of **m**_*p*_ = (1, 0, 0), STT tends to align spins in the free layer with **m**_*p*_ when 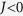 or with −**m**_*p*_ when *J* > 0. Thus it is expected that spin configuration of the free layer should change from the perpendicularly magnetized single domain at zero current density to an in-plane magnetized single domain at high current density at which the STT dominates the magnetic anisotropy and DMI. At an intermediate current density, the competition among STT, magnetic anisotropy and DMI may result in more complicated spin textures. Figure 2 shows the stable spin textures under various current densities, where the current is turned on throughout the simulations. Indeed, when current density changes from −5 × 10^12^ A/m^2^ (electrons flow along +*z*-direction) to 3 × 10^12^ A/m^2^, *m*_*z*_ experiences two step jumps around *J* = −2 × 10^12^ A/m^2^ and 1.4 × 10^12^ A/m^2^ while *m*_*x*_ varies steadily from 1 to −1 as shown in Fig. 2(a). For 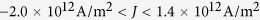, the spin configurations of the free layer are single domains with **m** almost perpendicular to the layer as shown in Fig. 2(d) at *J* = 0 and Fig. 2(e) at *J* = 0.4 × 10^12^ A/m^2^. For −4.4 × 10^12^ A/m^2^ < *J* ≤ −2 × 10^12^ A/m^2^ and 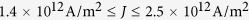, the single domain becomes a Neel wall as shown in Fig. 2(c,f) at *J* = 2.2 × 10^12^ A/m^2^ and *J* = 1.6 × 10^12^ A/m^2^, respectively. Here the chirality of a Neel wall is defined as the spin winding direction when one moves from the left domain to the right one, positive for clockwise winding and negative for counterclockwise winding. The chirality is uniquely determined by the sign of *D*. For *D* > 0 considered here, the chirality of a Neel wall is always counterclockwise^21^. The polarity of the Neel wall (*p* = ±1 if the magnetization in the centre of a wall points toward ±*x* direction) is 1 (−1) for *J* < 0 (*J* > 0). For *J* < −4.4 × 10^12^ A/m^2^ and *J* > 2.5 × 10^12^ A/m^2^, the ground state of the free layer is single domain with spins align along either **m**_*p*_ (for *J* < 0) or −**m**_*p*_ (for *J* > 0) as shown in Fig. 2(b,g) at *J* = −4.8 × 10^12^ A/m^2^ and *J* = 3.0 × 10^12^ A/m^2^, respectively. The asymmetric behaviour of the spin configuration in current density *J* comes from the asymmetry of the Slonczewski torque. It is the transmitted electrons that exert a STT on the free layer when electrons flow from the fixed layer to the free layer. In contrast, it is the reflected (by the fixed layer) electrons that exert a STT on the free layer when current direction is reversed. Thus, the two processes are not the same (asymmetric).

**Figure 3:**
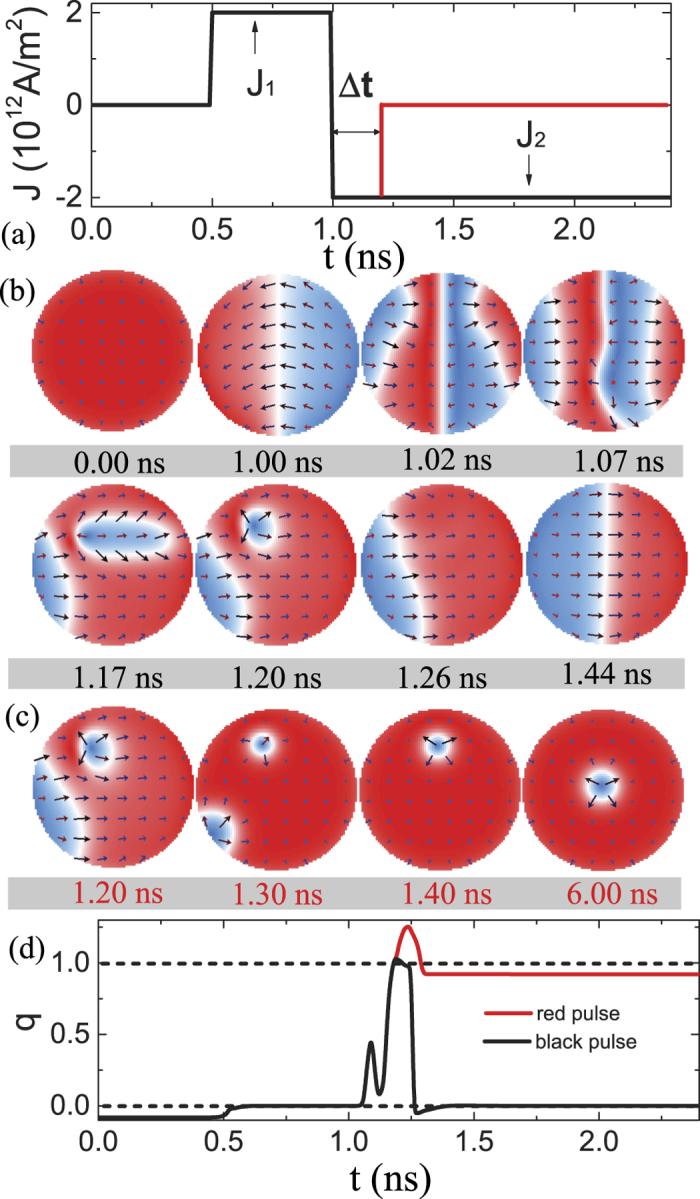
Nano-second current pulse for skyrmion creation. (**a**) Two nano-second current pulses: Black pulse (a current of *J*_1_ = 2 × 10^12^ A/m^2^ is on for 0.5 ns, followed by *J*_2_ = −2 × 10^12^ A/m^2^) is capable of changing a single domain to a Neel wall then to the image inversion (**m** → −**m**) of the Neel wall. Red pulse (a current of *J*_1_ = 2 × 10^12^ A/m^2^ is on for 0.5 ns, followed by *J*_2_ = −2 × 10^12^ A/m^2^ for 0.2 ns before switching to zero) is capable of creating a skyrmion from a Neel wall. Δ*t* is the duration of *J*_2_. (**b**) Snapshots of spin configurations at various times when the black pulse is applied. (**c**) Snapshots of spin configurations at various times when the red pulse is applied. A stable skyrmion is obtained. The color bar is the same as that in Fig. 2. (**d**) Skyrmion number as a function of time for black pulse and red pulse shown in Fig. 3a. The dashed lines indicate the position of *q* = 0 and 1,respectively.

Consider the connection and difference between Neel walls and skyrmions discussed early, we would like to see whether skyrmions can appear when the free layer changes from one type of Neel wall to the other. As an example, we first apply a current of *J*_1_ = 0.4 × 10^12^ A/m^2^ at 0.5 ns for about 0.5 ns, see Fig. 3(a), to create a Neel wall (spin configuration at 1 ns in Fig. 3(b)) from a single domain (spin configuration 0 ns in Fig. 3(b)). Then the current direction is reversed at *t* = 1 ns, as shown by the black line in Fig. 3(a). The Neel wall obtained at 1 ns changes eventually to its inversion (**m** → −**m**) (spin configuration at 1.44 ns in Fig. 3(b)), which is stable at current density of *J*_2_ = −2.0 × 10^12^ A/m^2^. On its way from one Neel wall to its inversion, the spin configuration undergoes a complicated transformation as shown by the snapshots at *t* = 1.02 ns; 1.07 ns; 1.17 ns; 1.20 ns; and 1.26 ns in Fig. 3(b). Interestingly, a hedgehog skyrmion appears at 1.17 ns and 1.20 ns. Thus, one expects that the hedgehog skyrmion will survive if current is switched off at 1.2 ns as shown by the red line in Fig. 3(a). Figure 3(c) shows the snapshots of the spin configurations at various times, *t* = 1.2 ns; 1.3 ns; 1.4 ns. The final stable spin configuration is indeed a hedgehog skyrmion represented by the snapshot at 6 ns. From the topological viewpoint, Fig. 3(d) is the time evolution of the skyrmion number for two current pulses denoted by black and red lines in Fig. 3(a). The skyrmion number for the black-line pulse changes from −0.08 for the initial domain, jumps to around 0.5 around 1.1 ns, and further rises to around 1 between 1.16 ns and 1.25 ns, then falls down to 0 at 1.35 ns when the free layer is in the Neel wall state. For the red-line pulse in Fig. 3(a), the time evolution of the skyrmion number is the same before 1.2 ns and stays around 1 after 1 ns, as expected. It should be pointed out that non-integer skyrmion number in Fig. 3(d) is due to finite size, and it should be always an integer number in the thermodynamical limit of an infinite system without boundary^13^.

**Figure 4:**
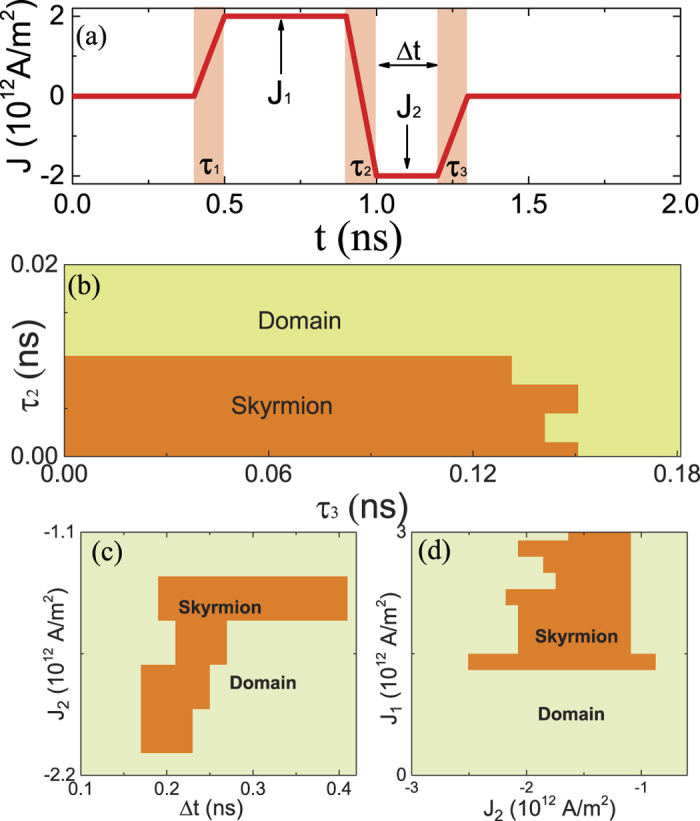
Phase diagram for skyrmion creation. (**a**) The illustration of the current pulse with current rising/falling time *τ*_1_, *τ*_2_ and *τ*_3_. (**b**) The phase diagram of skyrmion creation in *τ*_2_ − *τ*_3_ plane at Δ*t* = 0.2 ns, *J*_1_ = 2 × 10^12^ A/m^2^ and *J*_2_ = −2 × 10^12^ A/m^2^. (**c**) Phase diagram in *J*_2_ − Δ*t* plane for a fixed *J*_1_ = 2 × 10^12^ A/m^2^. (**d**) Phase diagram in *J*_2_ − *J*_1_ plane for Δ*t* ≈ 0.2. The orange region denotes the skyrmion state while the light green is for the single domain state.

We also consider the influence of the rise/fall time of current pulse *J*_1_ and *J*_2_ on the skyrmion generation and show the results in Fig. 4(a,b). As the role of pulse *J*_1_ is to generate a Neel wall, the final state only depends on the magnitude of current density *J*_1_ and is insensitive to the rise time of pulse *J*_1_ (*τ*_1_). Here the main influences to the skyrmion generation are the *J*_1_ − *J*_2_ transition time (*τ*_2_) and the fall time of pulse *J*_2_ (*τ*_3_). Figure 4(b) is the phase diagram in *τ*_2_ − *τ*_3_ plane. To guarantee a skyrmion generation, *τ*_2_ should be smaller than 0.012 ns while *τ*_3_ should be smaller than 0.15 ns. Moreover, the current densities *J*_1_, *J*_2_, and the duration time Δ*t* of *J*_2_ should be chosen properly in order to create hedgehog skyrmions. Figure 4(c) is the phase diagram in *J*_2_ _−_ Δτ plane at fixed *J*_1_ = 2.0 × 10^12^ A/m^2^. The orange region denotes the skyrmion phase while the light green denotes the domain phase. Use *J*_2_ = −1.8 × 10^12^ A/m^2^ as an example, a skyrmion can only form in the window 0.18 ns < Δ*t* < 0.24 ns. Figure 3(d) is the phase diagram in 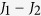 plane for 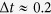 ns.

**Figure 5:**
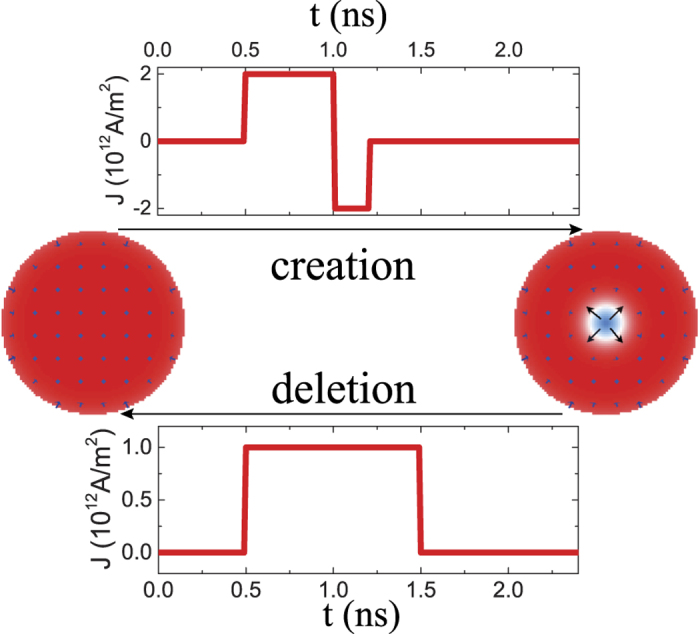
Switch between a skyrmion and a domain. Creation/deletion of a skyrmion from/to a single domain. The top current pulse is capable of creating a skyrmion while the bottom current pulse is capable of deleting a skyrmion. The color bar is the same as that in Fig. 2.

Obviously, one can also use a current pulse to delete a skyrmion. Within our model, a nano-second current pulse shown by the top curve of Fig. 5 can create a skyrmion from a single domain (indicated by the long arrow to the right) while another current pulse shown by the bottom curve of Fig. 5 can delete the skyrmion, and change the system back to a single domain state as indicated by the long arrow pointing to the left. We would like to point out that sub-nanosecond current pulses can be routinely created in laboratories around the world since they have been used to manipulate the magnetic structures in both nanostrips and spin valves^25^,^26^,^27^,^28^,^29^,^30^. Thus, it should not be hard to experimentally realize our method of skyrmion creation and manipulation. It is interesting to see the differences between our proposal and an early proposal^22^. Firstly, the other proposal requires both electric current and magnetic field while our proposal requires only the electric current. Secondly, the early method needs to use a very large current to create a ring-shape domain wall by nucleating a new domain while our proposal does not need to do so. Due to strong exchange field and anisotropy field in the bulk of a domain, the nucleation of a new domain requires very large electric current or magnetic field. For *D* = 3 mJ/m^2^, a current density as large as 5 × 10^12^ A/m^2^ cannot nucleate a new domain inside a single domain film^22^ while our method only needs the current density 1.3 × 10^12^ A/m^2^. This reduction of current density would significantly reduce the heating effect. Thirdly, transformation between skyrmions and Neel walls (or domains) in our proposal is reversible and reproducible. This may be preferable for writing and deleting information in a magnetic disk.

**Figure 6:**
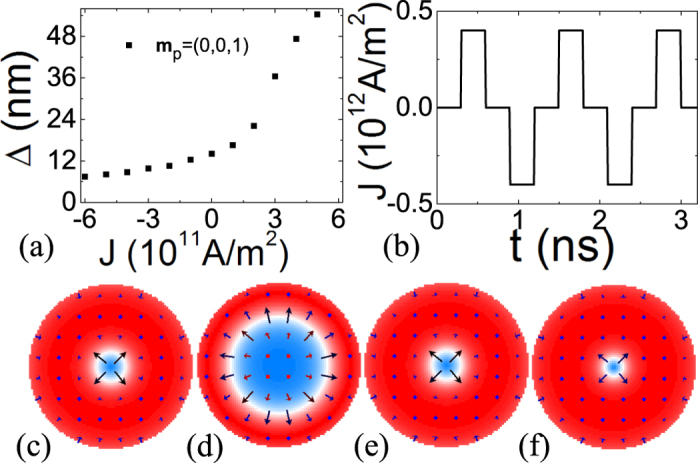
Manipulation of skyrmion size. (**a**) Skyrmion diameter as a function of current density. The core spins of the skyrmion point to +*z*-direction. (**b**) An applied current pulse. (**c**–**f** ) Spin configurations of the skyrmions at current density *J* = 0, 4 × 10^11^, 0, −4 × 10^11^ A/m^2^, respectively. The color bar is the same as Fig. 2.

Case of **m**_*p*_ = (0, 0, 1). In the case of **m**_*p*_ = (0, 0, 1), although skyrmion is a metastable state of the free layer, we do not find our simple skyrmion creation strategy working here although there should be, in principle, ways to create a skyrmion because both skyrmion and single domain are stable and/or metastable states. Nevertheless, the size of a preexistent skyrmion can be effectively tuned by a current as shown in Fig. 6. Figure 6(a) is the current density dependence of the skyrmion diameter. The size increases with current density for *J* > 0 and decreases for *J* < 0. The behaviour can be understood from the fact that the STT tends to align spins along −*z*-direction for *J* > 0 so that a skyrmion whose core spins are along −*z*-direction tends to expand. Hence the skyrmion size should increase as *J* increases. The spin configurations under various current densities shown in Fig. 6(b) are plotted in Fig. 6(c–f), and it shows clearly the sensitivity of skyrmion size to *J*.

In conclusion, nano-second current pulses can be used to create/delete skyrmions in a perpendicularly magnetized free layer of a spin valve when its fixed layer is in-plane magnetized. For a perpendicularly magnetized fixed layer, the size of a preexistent skyrmion in the free layer can be effectively controlled by a current.

## Additional Information

**How to cite this article**: Yuan, H. Y. and Wang, X. R. Skyrmion Creation and Manipulation by Nano-Second Current Pulses. *Sci. Rep.* **6**, 22638; doi: 10.1038/srep22638 (2016).

## Figures and Tables

**Figure 1:**
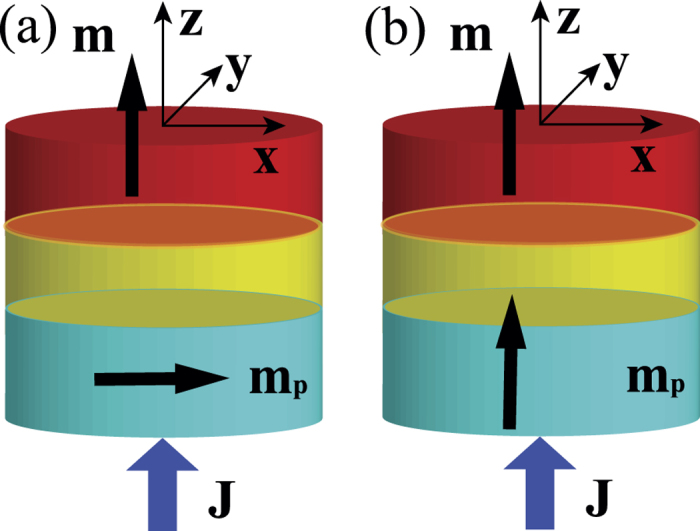
Spin valve structures. Spin valve with a free layer, a spacer layer and a fixed layer. **m** and **m**_*p*_ are respectively the unit vectors of the magnetization of free layer and the fixed layer. The layers are in the xy-plane, and current **J** flow along z-direction. **m**_*p*_ = (1, 0, 0) for (**a**) and **m**_*p*_ = (0, 0, 1) for (**b**).
