# Erratum: Skyrmion Creation and Manipulation by Nano-Second Current Pulses

**DOI:** 10.1038/srep34898

**Published:** 2016-10-10

**Authors:** H. Y. Yuan, X. R. Wang

Scientific Reports 6: Article number: 22638; 10.1038/srep22638 published online: 03 03 2016; updated: 10 10 2016.

This Article contains referencing errors. In the Introduction section,

“One type of such systems such as MnSi, Fe_1−x_Co_x_Si, Cu_2_OSeO_3_^3,4,5,6^ with the bulk Dzyaloshinskii-Moriya interaction (DMI), has chiral crystal structure that supports vortex-like skyrmions. Another type of the systems are heavy metal/ultrathin ferromagnetic layers such as Fe/Ir, Ni/Co and Ta/CoFeB/TaO_x_^10,11,28,29^, that results in an interfacial DMI”.

should read:

“One type of such systems such as MnSi, Fe_1−x_Co_x_Si, Cu_2_OSeO_3_^3,4,5,6^ with the bulk Dzyaloshinskii-Moriya interaction (DMI)^28,29^, has chiral crystal structure that supports vortex-like skyrmions. Another type of the systems are heavy metal/ultrathin ferromagnetic layers such as Fe/Ir, Ni/Co and Ta/CoFeB/TaO_x_^10,11^, that results in an interfacial DMI”.

In addition, in the Results and Discussion section,

“We would like to point out that sub-nanosecond current pulses can be routinely created in laboratories around the world since they have been used to manipulate the magnetic structures in both nanostrips and spin valves^25–30^”.

should read:

“We would like to point out that sub-nanosecond current pulses can be routinely created in laboratories around the world since they have been used to manipulate the magnetic structures in both nanostrips and spin valves^25–27^”.

In the Introduction section of the HTML version of the Article,

“It is noticed that the spin structure along the radial direction of a hedgehog skyrmion is very similar to that of a Neel wall^11^”.

should read:

“It is noticed that the spin structure along the radial direction of a hedgehog skyrmion is very similar to that of a Neel wall^30^”.

